# Occupational exposure in swine farm defines human skin and nasal microbiota

**DOI:** 10.3389/fmicb.2023.1117866

**Published:** 2023-03-29

**Authors:** Xiran Wang, Dongrui Chen, Juan Du, Ke Cheng, Chang Fang, Xiaoping Liao, Yahong Liu, Jian Sun, Xinlei Lian, Hao Ren

**Affiliations:** ^1^Guangdong Laboratory for Lingnan Modern Agriculture, National Risk Assessment Laboratory for Antimicrobial Resistance of Animal Original Bacteria, College of Veterinary Medicine, South China Agricultural University, Guangzhou, China; ^2^Guangdong Provincial Key Laboratory of Veterinary Pharmaceutics, Development and Safety Evaluation, South China Agricultural University, Guangzhou, China; ^3^Jiangsu Co-Innovation Center for the Prevention and Control of Important Animal Infectious Disease and Zoonoses, Yangzhou University, Yangzhou, China; ^4^Guangxi State Farms Yongxin Jinguang Animal Husbandry Group Co., Ltd, Nanning, China

**Keywords:** occupational exposure, human microbiota, microbial diversity, swine farm, longitudinal investigation

## Abstract

Anthropogenic environments take an active part in shaping the human microbiome. Herein, we studied skin and nasal microbiota dynamics in response to the exposure in confined and controlled swine farms to decipher the impact of occupational exposure on microbiome formation. The microbiota of volunteers was longitudinally profiled in a 9-months survey, in which the volunteers underwent occupational exposure during 3-month internships in swine farms. By high-throughput sequencing, we showed that occupational exposure compositionally and functionally reshaped the volunteers’ skin and nasal microbiota. The exposure in farm A reduced the microbial diversity of skin and nasal microbiota, whereas the microbiota of skin and nose increased after exposure in farm B. The exposure in different farms resulted in compositionally different microbial patterns, as the abundance of Actinobacteria sharply increased at expense of Firmicutes after exposure in farm A, yet Proteobacteria became the most predominant in the volunteers in farm B. The remodeled microbiota composition due to exposure in farm A appeared to stall and persist, whereas the microbiota of volunteers in farm B showed better resilience to revert to the pre-exposure state within 9 months after the exposure. Several metabolic pathways, for example, the styrene, aminobenzoate, and N-glycan biosynthesis, were significantly altered through our PICRUSt analysis, and notably, the function of beta-lactam resistance was predicted to enrich after exposure in farm A yet decrease in farm B. We proposed that the differently modified microbiota patterns might be coordinated by microbial and non-microbial factors in different swine farms, which were always environment-specific. This study highlights the active role of occupational exposure in defining the skin and nasal microbiota and sheds light on the dynamics of microbial patterns in response to environmental conversion.

## Introduction

Human microbiome generally refers to the trillions of symbiotic microorganisms that reside in each person, comprised of a variety of bacteria (Lloyd-Price et al., 2016), fungi (van Tilburg Bernardes et al., 2020), archaea (Kim et al., 2020), and viruses (Ursell et al., 2012; Liang and Bushman, 2021). The microbiomes extensively interact with their hosts by offering genetic diversity (Hall et al., 2017), essential determinants of immunity (Kamada et al., 2013), and functional entities that modulate the nutrients and drug metabolisms (Rooks and Garrett, 2016). There have been plenty of studies that defined how bacterial functionality and diversity shaped their hosts from various key aspects. For instance, Henry et al. s illustrated that the colonized microbiome was able to influence the host’s evolutionary potential by extending the host’s genetic repertoires (Henry et al., 2021). A recent study also identified how by priming the activation of cGAS-STING signaling, gut microbiota plays a significant role in host antiviral immunity (Erttmann et al., 2022). In another study, Perxachs et al. reported that host depression severity was directly associated with proline, whose circulation was dependent on microbiome composition and functionality (Mayneris-Perxachs et al., 2022). These data and others collectively shed light on the immense impact of the microbiome on host traits, which is now considered as the ‘Second Genome’ of the host (Grice and Segre, 2012). Despite contribution of microbiome on host traits, the host is also reportedly able to influence the microbiome (Pirr and Viemann, 2020). Previous studies indicated that the host taxa and phylogeny appear as one dominant determinant on the assemblage of bacterial communities (Roeselers et al., 2011; Tzeng et al., 2015). Other host specific factors, such as temperature (Kokou et al., 2018), moisture (Grice and Segre, 2011), and pH (Kwon and Lee, 2022), are, in the given niches, able to exert impact on shaping the microbiome, but the exact influence of these factors differs according to different studies (Lemieux-Labonté et al., 2016).

From the aforementioned examples, it is pronounced that microbiomes shape their hosts and vice versa. However, except for those host intrinsic factors, more and more evidence supports the idea that the microbiome of animals, including humans, highly responds to environmental cues (Lavrinienko et al., 2018). Both microbial and non-microbial factors in the environment may be involved in shaping the microbiome of the host *via* direct or indirect contact. This has been comprehensively summarized in a previously-published review (Ahn and Hayes, 2021). In this review article, Ahn et al. conclusively addressed macroenvironmental factors such as the chemical environment, built environment, and socioeconomic environment, and the microbiome modifications therein. Among these macroenvironmental factors, work-related environmental exposures, namely, occupational exposure, are often in higher orders of magnitude compared to daily life exposure (Lai and Christiani, 2019). On the one hand, microbial exposures were enriched in certain occupational conditions, e.g., textile mills or livestock farms, and are reported to promote the microbial exchange between humans and the surroundings (Olenchock et al., 1983; Schierl et al., 2007). One direct evidence was that the animal caretakers were colonized by strain-specific methicillin-resistant *Staphylococcus aureus* (MRSA) present in their work environment (Frana et al., 2013; Fang et al., 2014). On the other hand, non-microbial exposures, such as aerosols (Pan et al., 2021), chemicals (Tian et al., 2020), metals, and particles (Lear et al., 2021), in working spaces have been recognized as changing the human microbiome (National Academies of Sciences and Medicine, 2018). A prior study indicated that farmers who were exposed to pesticides generally presented a shifted microbiome and reduced microbial diversity in comparison with those who were not exposed (Stanaway et al., 2017). Regarding the work-related shifts in the microbiome, tremendous effort has been made on deciphering the effect on microbiota in the gut; however, recent studies highlighted that the microbiota on skin and respiratory tracts also deserve special attention as the skin represents the largest organ and is frequently under exposure to external environments (Kim et al., 2018). The skin microbiota has been fundamentally overlooked, possibly due to bias from the growth of microbes in artificial settings (Sandhu et al., 2019). Furthermore, a recent meta-analysis concluded that, in some cases, the skin microbiome could be a better indicator than the gut microbiome for the prediction of host physiology, but to date, there has been a lack of focus of research on the skin and the respiratory tract (Huang et al., 2020). Given that skin/nasal microbiota are generally more vulnerable to many extrinsic factors, a major knowledge gap remains regarding the specific responses and actions of these microbiota to environmental shifts (Boxberger et al., 2021; Larson et al., 2022). Lamentably, there is insufficient research and, therefore, a lack of an in-depth understanding of the interactions between skin/nasal microbiota and occupational exposure across different conditions and hosts (Lemieux-Labonté et al., 2016).

Previously, we performed a longitudinal study to depict the temporal changes in the gut microbiome and resistome under occupational exposure in swine farms (Sun et al., 2020). The results indicated that even acute changes in the working environment induced profound remodeling effects on the gut microbiome and resistome. One limitation of this study was that the potential changes in skin and nasal microbiota were not further investigated. Therefore, we further this scope in the present study to decipher the dynamic changes of skin and nasal microbiota under occupational exposure in swine farms. With this in mind, we traced the skin and nasal microbiota alteration of nine trainee students during and after a 3-month internship and found that temporary occupational exposure can reshape the skin and nasal microbiota, yet the modification is always environment-specific. This study highlights the microbial patterns of skin and nose formed by occupational exposure and explains the possible interaction between microbiota and the environment.

## Materials and methods

### Study design

A total of 10 student were originally recruited for this study; men with an average age of 24 years, and the following student IDs: A, B, C, D, E, F, G, H, I, and J. However, student E asked to quit during the first week of practice due to a personal health problem; thereafter he was omitted from further analysis. Written informed consent was provided by all students voluntarily. From July 2016 to June 2017, all students were randomly divided into two groups, A and B, with four volunteers and five volunteers, respectively. They were assigned to two different swine farms for veterinary clinical practice as required by the veterinary medicine major training program at South China Agricultural University. The two sampling farms were in the two provinces that account for the major pork production in China: Hunan and Guangdong. A survey was performed in advance of the sampling to compare the breeding scale, management model, functional division, and operation time of the different farms. Both farms were highly similar in their use of medication (penicillin, amoxicillin, gentamicin, florfenicol, enrofloxacin, sulfonamides, etc.,) and were selected as candidates for representing the general environments of swine farms in China.

### Sample collection and preparation

Three phases, including eight-time points, were sampled as T0 (1–2 weeks before arriving at the farm), T1-3 (collecting samples monthly for the consecutive 3 months at the farm), and T4-9 (collecting samples monthly for another 3 months and on the ninth month after the students had been practicing at the farm). All samples in this study were collected at the time the volunteers returned to the living area from the swine farm working area.

The facial skin, which is often exposed to the external environment, was selected to present the skin issue. To completely collect the facial skin samples from the volunteers’ foreheads, sterile cotton swabs were soaked in saline, pressed, and forced to bend 45 degrees, before wiping the sampling surface (4 cm^2^) smoothly and slowly for 30 s. Then, the other end of the swab was used to wipe the skin along the vertical direction of the previous wiping. Following wiping, the swabs were put into sterile frozen tubes. The nasal swab samples were collected by gently wiping the surface of the nasal mucosa with a sterile cotton swab soaked in saline. Each nasal mucosa was wiped twice in the same area with the same swab. After wiping, the cotton swab was kept in sterile frozen tubes and rotated. Both facial skin and nasal samples were kept on dry ice and immediately transferred to the laboratory, where they were stored at −80°C for further DNA extraction.

### DNA extraction and quality control

The samples used in our study were collected from each volunteer at consecutive time points, extracted, and sequenced separately. Bacteria genomic DNA was isolated from skin and nasal samples using the HiPure Stool DNA Kit (Magen, No. D3141) according to the manufacturer’s instructions. The brief procedures were described in our previous study (Sun et al., 2020). The extracted DNA was eluted in sterile water and the quality was evaluated by NanoDrop 2000 Spectrophotometer (Thermo Fisher Scientific, United States).

### 16 s rRNA amplification and sequencing

The composition of bacterial communities in skin and nasal samples was determined by amplification and analysis of the V3-V4 hypervariable region (forward primer 338F, 5’-ACTCCTACGGGAGGCAGCAGG-3′; reverse primer 806R, 5’-GGACTACHVGGGTWTCTAAT-3′) of the 16S rRNA gene with polymerase chain reaction (PCR). The primers’ synthesis was completed by Major Biosystem Corporation (Shanghai, China). After two rounds of PCR amplification, the products were purified and the paired-end indexes and adapters were added to the ends of the amplicons for sequencing libraries construction. After quality control, the purified libraries were sequenced with the Illumina MiSeq platform with paired-end reads 300 base pairs (bp) in length.

### Preprocessing of 16 s rRNA amplification sequencing data

Bioinformatic analysis was implemented using the Quantitative Insights into Microbial Ecology QIIME2 (version 2020.11)^1^ platform. The quality control of raw Illumina amplicon sequence data was processed using the DADA2 algorithm, removing the chimeric sequences, and truncating the sequences from 1 to 290 bases of the forward reads, and from 1 to 270 bases of the reverse reads. The representative sequences Features were obtained in the process of Denoise. Rarefaction curves show the number of Features constructed at different sampling depths. These curves can make a preliminary assessment of sequencing saturation. If the final curve tends to level, it means that the current sequencing saturation is sufficient. Rarefaction curves were generated by the QIIME2 pipeline (q2-diversity plugin). Sequences were rarefied at 10 sequencing depths in order to best visualize the change in diversity with respect to sampling depth. Features taxonomic classification annotation was carried out by the q2-feature-classifier plugin while using a downloaded classifier made by the species annotation reference database SILVA (version 138). Phylogenetic diversity analyses were achieved *via* the q2-phylogeny plugin, which used the MAFFT program to perform multiple sequence alignments on the representative sequences (FeatureData in QIIME2) and the FastTree program to generate a phylogenetic tree from the alignments. The Shannon entropy and Chao1 were used to evaluate alpha diversity, all of these indices were calculated by the QIIME2 pipeline (q2-diversity plugin). Functional composition profiles of the skin and nasal microbiota of volunteers before, during and after the exposure were predicted from the 16S rDNA gene sequences using PICRUSt2 (version 2.3.0_b) (Phylogenetic Investigation of Communities by Reconstruction of Unobserved States) with KEGG (Kyoto Encyclopedia of Genes and Genomes) database pathways. LEfSe (linear discriminant analysis effect size) analyses were analyzed on the Galaxy website^2^ (version 1.0) for the characteristic microbial taxa on genus level in three phases (Segata et al., 2011).

### Statistical analysis

In order to concisely and comprehensively demonstrate the data, the eight samplings were assigned to three stages that represented the time before, during, and post the exposure. Statistical analysis was implemented with the R platform (version 4.1.2, R Core Team 2021). Statistical differences in Bray-Curtis distances between groups were calculated with the “vegan” package to evaluate beta diversity and were tested using analysis of similarity (ANOSIM, available through the “vegan” package) by permutation of group membership with 999 replicates. Principal coordinate analysis (PCoA) was performed using the “ape” package based on the Bray-Curtis distances and visualized using the “ggplot2” package. To reveal the abundance change during the three phases, a *t*-test was performed based on the genus’ relative abundance. We also calculated *p*-values and log_2_FoldChange to plot the volcano plot with the package “ggplot2”. In order to reveal the abundance change of the KEGG pathway, results were predicted from the 16S rDNA gene sequences using PICRUSt2 software during the three phases. The *t*-tests were conducted for each group and the extended error bar charts, composed of the bar chart showing the average value of functional abundance between groups on the left and the scatter chart showing the average abundance and its 95% confidence interval between groups on the right, were drawn with the package ‘ggplot2’ to show the results. In order to show the dynamic changes in bacterial composition on phyla and genus levels during the three phases, the t-tests were performed based on relative abundance. The means, standard deviation, *p*-values, and adjusted *p*-values (FDR, false discovery rate correction) were calculated, and the pie charts of results on phyla level were plotted with the function “pie”.

## Results

### Amplicon sequencing and quality control

**Figure 1:**
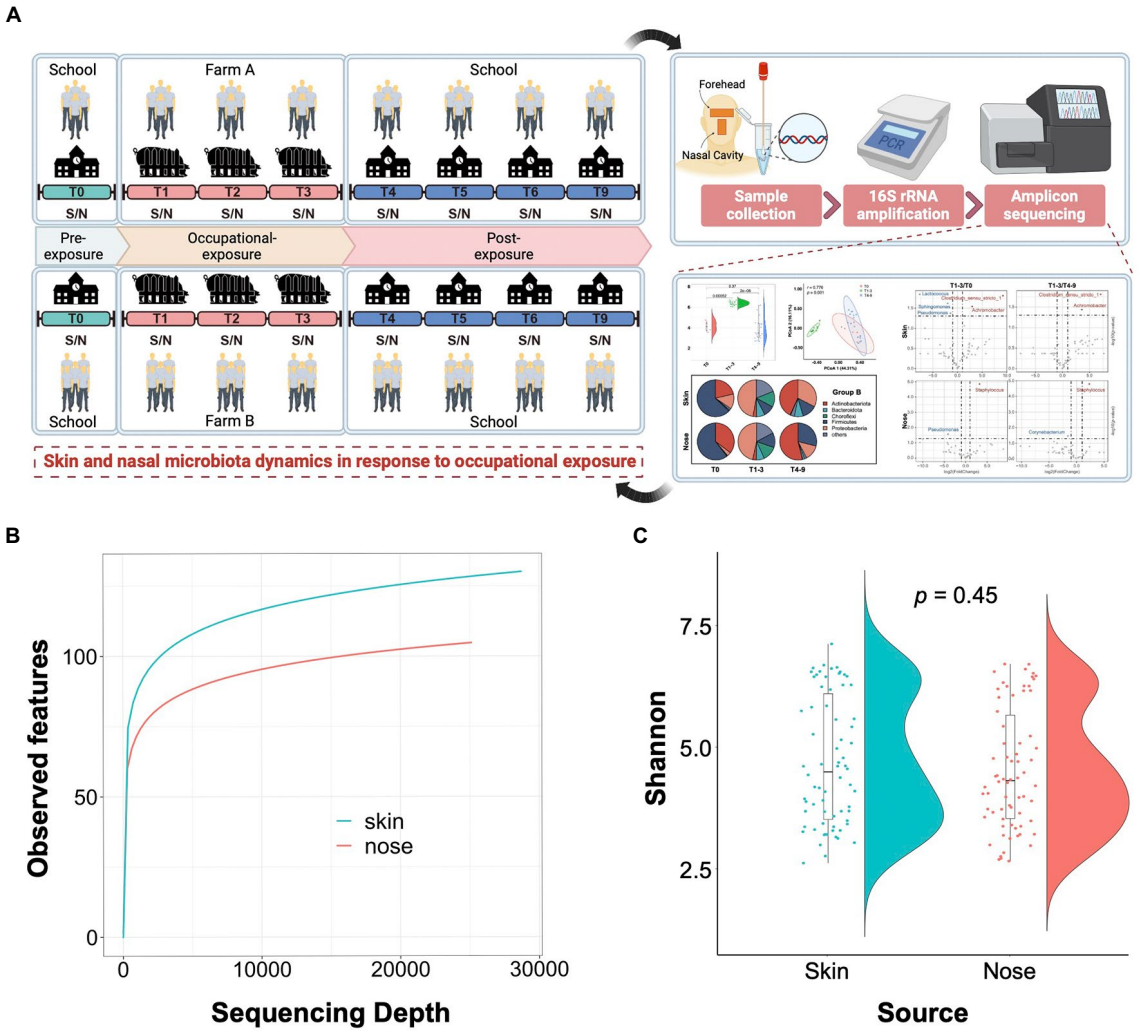
Study design and quality control of amplicon sequencing data. **(A)** Schematic overview of the study design. S/N stands for the skin and nasal samples collected from the foreheads and nasal cavities of volunteer students. **(B)** Alpha rarefaction curves show a relatively higher bacterial community richness in the skin samples compared to the nasal samples. The average number of the observed features for each sequencing depth is presented. **(C)** Shannon entropy for skin and nasal swab samples. The t-tests were conducted to compare the diversity between skin and nasal swab samples.

As illustrated in Figure 1A, a 9-month longitudinal survey was conducted on 9 students to decipher the dynamic alteration in the skin and nasal microbiota before and after occupational exposure in swine farms. By sequencing the 140 collected samples, a total of 5,846,711 16S rRNA sequences were obtained with an average of 41,762 16S rRNA sequences per sample. The sequences generated from skin samples ranged from 3,0092 to 86,561 and 3,0007 to 87,814 from nasal samples. Compared with the nasal cavities, the skin generally harbored a bacterial community with higher richness, indicated by the alpha rarefaction curves. The curves became flat into a saturated-like state along with the increasing sample sizes, suggesting sequencing depth covered all species and sequencing data were qualified enough for further analysis (Figure 1B). The Shannon entropy showed that no significant difference was observed among skin and nasal samples regarding the alpha diversity (*p* = 0.45, Figure 1C).

### Occupational exposure modified microbial diversity of skin and nose in an environment-dependent manner

**Figure 2:**
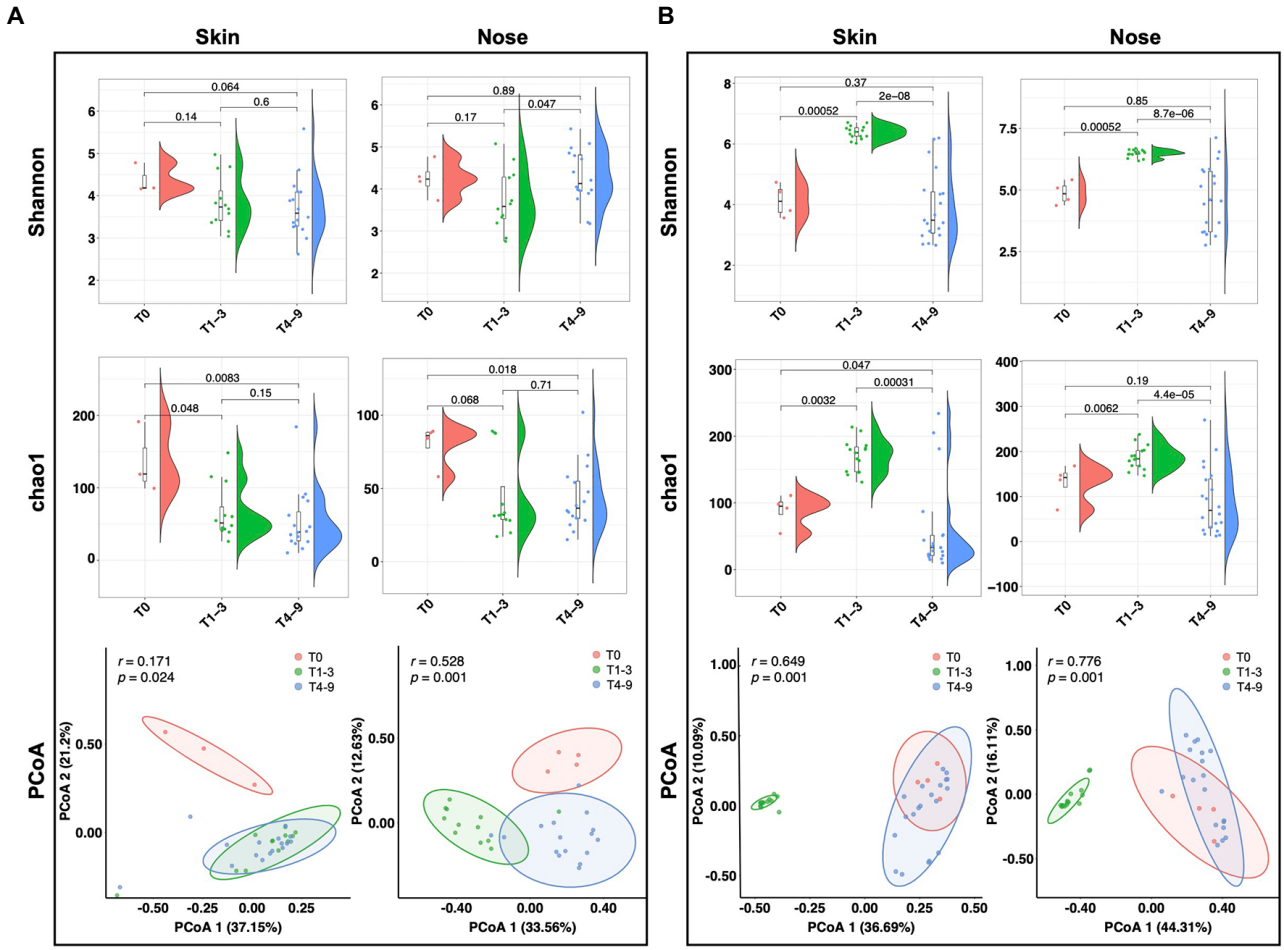
Microbial diversity alteration across three stages. Alpha and beta diversity of microbiota from students’ skin and nasal swab samples displayed in **(A)** group A and **(B)** group B. The Wilcoxon tests were conducted across different stages to display the significance of alpha diversity. The PCoA analysis was performed based on Bray-Curtis distances.

To understand the impact of occupational exposure on the skin and nasal microbial community, we first sought to estimate whether biodiversity was shifted by a stay in swine farms. Two alpha diversity indices, Chao1 and Shannon, were introduced to elucidate the bacterial abundance and evenness. For the students from group A, the Shannon index of both skin and nose reduced numerically but non-significantly after occupational exposure to a swine farm (T1-3 and T4-9). The Chao1 index either significantly reduced (*p* = 0.048) or showed a strong trend to reduce (*p* = 0.068) for skin and nasal microbiota after exposure in swine farm A (Figure 2A). In contrast, the occupational exposure in swine farm B significantly increased the microbial alpha diversity on skin and nose (T1-3, *p* < 0.01), yet this increased diversity was diminished after the interns returned the school (T4-9, *p* < 0.01, Figure 2B). Furthermore, the principal coordinate analysis (PCoA) was performed to address the beta diversity for explaining the inter-group variations. In swine farm A, all skin and nasal regimens collected after the exposure (T1-3 and T4-9) clustered together yet were distinguishable from the pre-exposure status (T0, Figure 2A). The different temporal patterns of microbiota diversity in farm B were observed, as the results supported that microbial structure of both skin and nose was discriminated in the swine farm (T1-3) compared to the pre- (T0) and post-exposure states (T4-9, Figure 2B). These results collectively confirmed that the work-related exposure modified microbial diversity in the skin and nasal cavity. However, we cannot generalize this effect since the modification majorly depended on the environment.

### Occupational exposure in swine farms compositionally remodeled the skin and nasal microbiota

**Figure 3:**
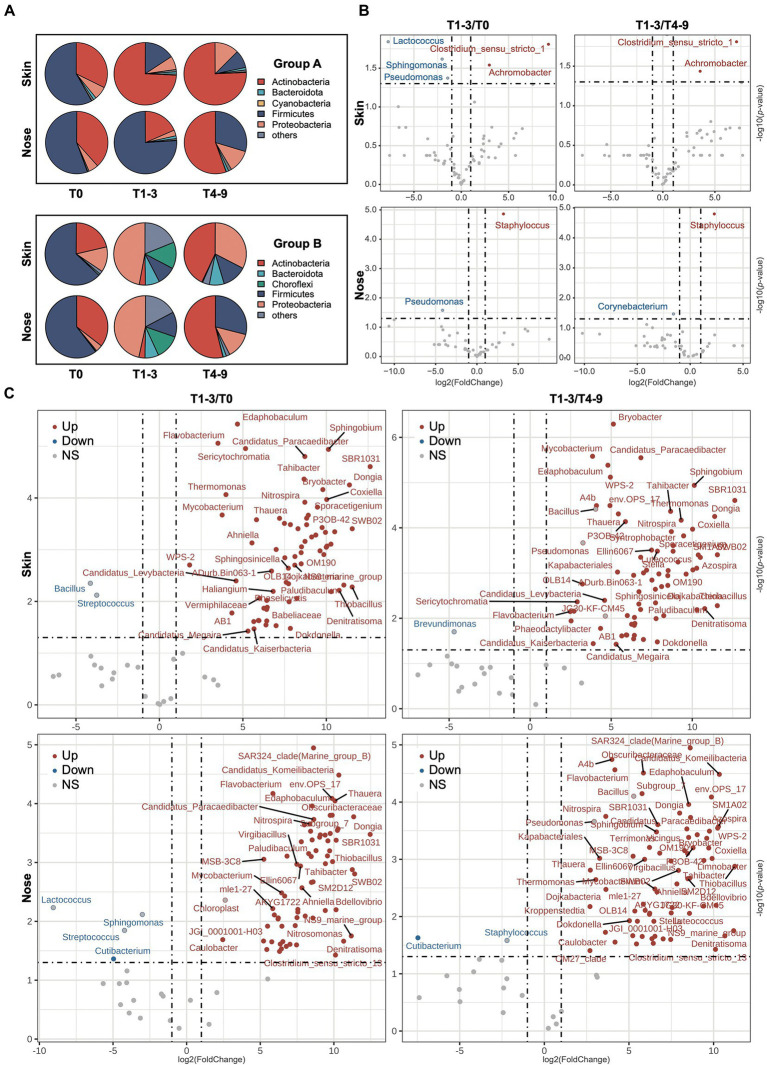
The variation of microbiota composition across stages. **(A)** The bacterial composition on the phyla level across stages is presented *via* pie charts. The top five phyla based on relative abundance from each stage were filtered and merged within two groups. The volcano plots show the alteration of microbiota composition on genus level of skin and nasal samples during three phases in **(B)** group A and **(C)** group B. The log2FoldChange was used to illustrate the variation of microbiota composition with occupational exposure in swine farms (T1-3) compared with T0 and T4-9. The red/blue dots represent the significant genera up−/downregulation in T1-3 compared to both T0 and T4-9, and the red−/blue-bordered, gray-filled dots indicate significant up−/downregulation in T1-3, compared to T0 or T4-9.

A total of 44 phyla, 253 orders, and 783 genera were assigned to the obtained sequences. A comprehensive overview of the taxonomic assignment was provided in Supplementary Tables S1–S4. As shown in the pie charts (Figure 3A), the skin and noses of students from both groups A and B were overarchingly dominated by the phylum Firmicutes before the occupational training, ranging from 60.33 to 66.31%. Other dominating phyla, including Actinobacteria and Proteobacteria, accounted for over 30% of all sequences obtained. After the exposure in the swine farms (T1-3), the students in farm A presented compositionally shifted microbiome on the skin as the relative abundance of Actinobacteria sharply increased at the expense of Firmicutes, whereas the nasal microbiota composition stayed relatively stable except for a slight increment of Bacteroidota abundance. Regarding the students in farm B, both skin and nasal microbiota were drastically altered, given that the Proteobacteria became the most predominant phylum in the skin and nasal samples collected from farm B and the abundance of Choroflexi phylum notably increased from less than 1% to over 12%. The abundance of Firmicutes increased but was not observed to restore dominance even after the students finished their occupational training in the swine farms (T4-9), suggesting the taxonomic changes in the skin and nasal microbial community appeared to stall and persist to some extent. Intriguingly, the Actinobacteria enriched among students from both A and B groups in the post-exposure state, even though this phylum increased to a significantly lesser extent in group B during T1-3.

To further probe the impact of occupational exposure on skin and nasal microbiota, the significantly shifted bacterial taxa were profiled at the genus level and shown with a volcano plot (Figures 3B,C). In farm A, the abundance of *Clostridium_sensu_stricto_1* and *Achromobacter* significantly increased in skin samples at timepoint T1-3 compared to both T0 and T4-9. *Staphylococcus*, which generally comprised a variety of topical pathogens, was particularly elevated in the nasal regimens of T1-3. The genus *Pseudomonas* eminently decreased in the skin and nasal regimens after the exposure but was found not significantly changed after the students had left the farm. As to farm B, the microbial alteration was more dynamic, as 162 genera were found significantly altered at timepoint T1-3 compared to T0 or T4-9 (Supplementary Table S5). As illustrated in Figure 3C, there were more increased than decreased genera in students who were exposed to farm B. This was consistent with the higher microbial diversity we observed in the section above. Specifically, we observed a moderate increment in several taxa such as *Flavobacterium*, *Mycobacterium*, *Denitratisoma*, and *Thauera*, which include many zoonotic pathogens. Among the few decreased genera, the relative abundance of *Streptococcus* was noted to significantly decrease in skin and nose during the stay in farm B, and the genus *Cutibacterium* was decreased during the time at the farm compared to pre−/post-exposure from nasal samples but not from skin samples. The result of microbiota alteration based on LEfSe analysis can be found in Supplementary Figure S1, as many taxa were regarded as biomarkers that appeared in both skin and nasal samples, which was consistent with the analysis of the abovementioned results. The microbiota dynamic of individuals in groups A and B was found to be impacted by occupational exposure (Supplementary Figure S2), which was consistent with our analysis using pooled data, thereby supporting the remodeling effect of occupational exposure on the microbiome.

### Microbial functional profiles were predicted to change in response to occupational exposure in swine farms

**Figure 4:**
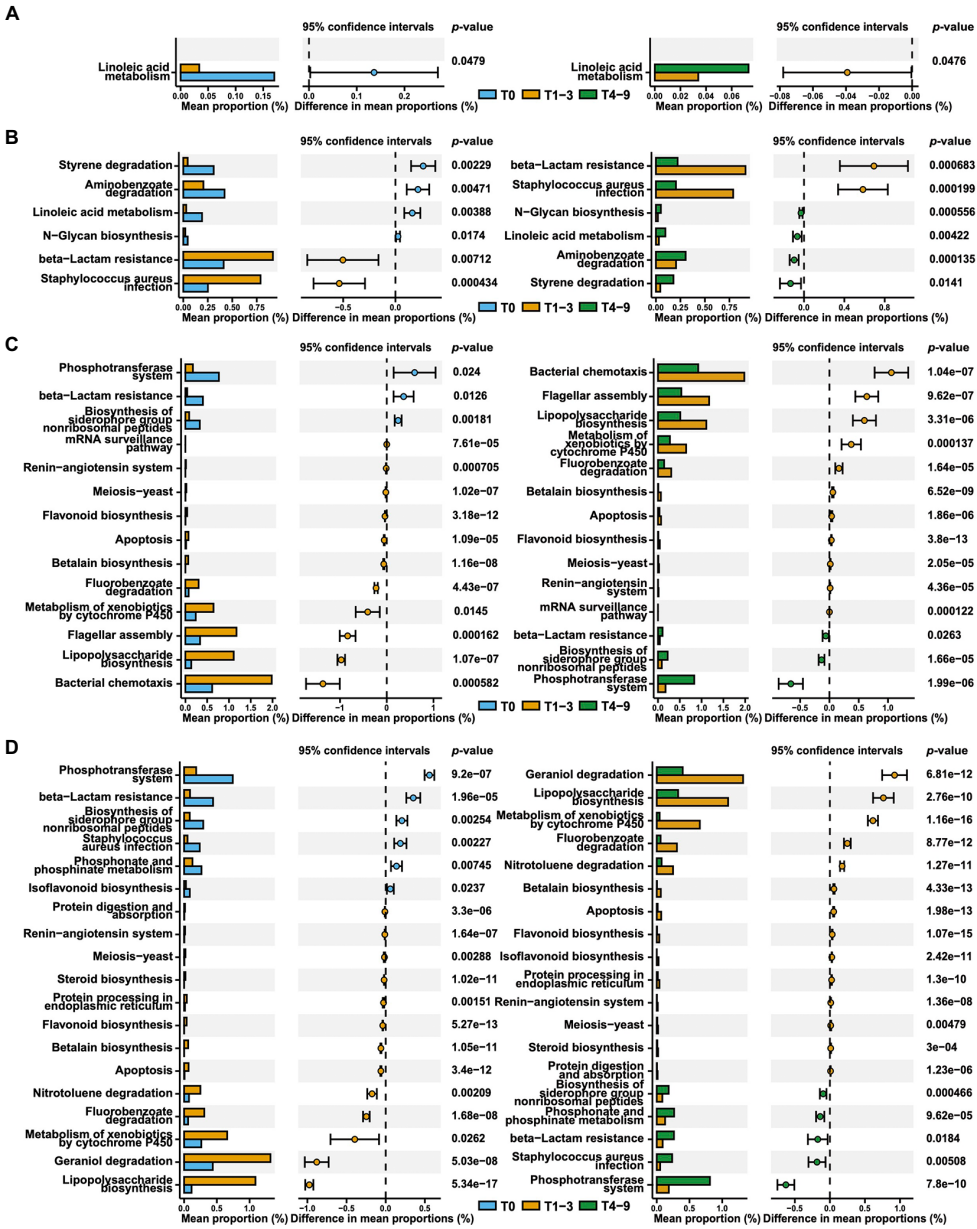
Predicted microbial function comparison across stages based on KEGG level-3. The skin **(A)** and nasal **(B)** swab results from group A and the skin **(C)** and nasal **(D)** swab results from group B across stages are presented *via* extended error bar charts. The two-sided t-test was conducted and the functions that significantly up−/downregulated in T1-3 compared to both T0 and T4-9 are presented.

The effects of occupational exposure in swine farms on functional pathways of skin and nasal microbiota were assessed by performing functional predictions analysis in PICRUSt2 using the KEGG orthology (KO) database (Figure 4). Compared to T0 or T4-9, there were a total of 582 predicted functions based on KEGG level-3 that significantly up−/down-regulated in response to occupational exposure in swine farms (Supplementary Table S7). For the students in farm A, the linoleic acid metabolism pathway was the only pathway that registered significant alterations in skin microbiota. Our PICRUSt analysis demonstrated that occupational exposure in farm A substantially weakened the metabolic activity of skin bacteria on linoleic acid in comparison with pre- and post-exposure states (T1-3 and T4-9). This pathway was also observed to be downregulated in nasal microbial communities of students in farm A, in concert with several other metabolic pathways including styrene, aminobenzoate, and N-glycan metabolisms. It is worth mentioning that the beta-lactam resistance was predicted to be enriched in the nasal microbiota in the case of exposure in farm A. As for the students in farm B, pathways involving the phosphotransferase system and biosynthesis of siderophore were reduced in both skin and nasal microbiota, whereas the functions involved in bacteria motility, such as chemotaxis or flagellar assembly, were significantly enhanced. However, it was interesting that the beta-lactam resistance was predicted to decrease during exposure in farm B, which was opposite to what we observed in farm A.

## Discussion

### Livestock farms as ideal targets to elaborate microbiome responses to environmental exposures

The skin, the largest organ of the body, and the nose offer a variety of niches for microbial communities (Manus et al., 2017; Kumpitsch et al., 2019). Due to their direct exposure to the external environment, the skin and nasal microbiomes are considered more dynamic than those in the gut (Romano-Bertrand et al., 2015). In the present study, the swine farm was selected to investigate the impact of occupational exposure on the skin and nasal microbiota. The scientific rationale for a focus on occupational exposure in the livestock industry is primarily based on the following reasons. The first reason is that animal husbandry represents an intermediate interface between the natural environment and human beings (Vanotti et al., 2017). It is of high interest to elucidate how animal husbandry bridges the gap between the environmental and human microbiome. Previous studies spotlighted that, *via* animal products and wastes, frequent microbial exchange between the environment and humans occurred as a result of complex interplays between the environment, animals, and humans (Téllez et al., 2015; Wang et al., 2021). As a result, antibiotic resistance genes (ARGs) of clinical importance might spread in tandem with such microbial exchanges, thereby posing a direct threat to public health (Xu et al., 2022). The second reason is that both microbial and non-microbial factors for occupational exposures are enriched in livestock farming. The rich microbial species in poultry, swine, and cattle farming mainly act as the microbial factors of occupational exposure (McFall-Ngai et al., 2013; de Rooij et al., 2019), with the skin and nasal microbiome of farmworkers possibly being easily affected by the environmental microorganisms *via* direct contact with animals, bedding materials, feeding facilities, and even airborne microbial communities (Kraemer et al., 2018; Moor et al., 2021; Yang et al., 2021). In addition, livestock farming generally requires high hygiene standards to maximally avoid economic losses caused by infectious diseases. It calls for intensive usage of disinfectants, antimicrobials, and antiseptics, which possibly drive the modification of human microbiome patterns (Martelli et al., 2017; Lim et al., 2020; Law et al., 2021). Taking these into account, swine farms might be the ideal locations for exploring the microbiome dynamic in response to occupational exposures. The present study majorly focused on the samples from volunteers to analyze the skin and nasal microbiome responses to environmental cues in swine farms. Future studies that include data from samples from environments and residing animals are necessary to comprehensively depict the kinetic and mechanism of microbiome formation in response to certain occupational exposures.

### Microbial and non-microbial factors in specific environments collectively coordinated the microbiome shift in response to occupational exposure

In this study, we collected samples from students who stayed on two swine farms with different geographical locations but with similar scales and working conditions. Interestingly, the skin and nasal microbiomes of the volunteers from the two farms demonstrated significantly different patterns. In farm A, the microbial diversity of the students sharply decreased after occupational exposure. This was different from what has been reported in most of the literature, where microbial diversities were generally observed to increase after exposure to livestock farms (Kraemer et al., 2021; Moor et al., 2021). Further analyses revealed that the observed changes in microbial composition and diversity were not resilient throughout the period, as they reverted after the students left the farms. We assumed that the decline in diversity and stalled compositional change were possibly related to specific non-microbial factors in farm A, such as strict hygiene practices. Due to the prevalence of certain veterinary pathogens, such as porcine reproductive and respiratory syndrome virus (PRRSV), African swine fever virus (ASFV), *Mycoplasma hyopneumoniae*, and *Streptococcus suis* in China, some swine farms always require practitioners to take a series of sanitation procedures (He et al., 2011; Li et al., 2014; Wang et al., 2018; Dong et al., 2021). The direct exposure of skin and nose to topical decontaminants exerts strong selection pressure on the microbiome, which likely resulted in reduced diversity. Not surprisingly, the microbiomes after decontaminant selection compositionally encompass a range of bacterial persisters with strong colonization capability, which partially explained the suboptimal resilience of the microbiome after occupational exposure in farm A. In contrast to farm A, the skin and noses of volunteers in farm B were detected with elevated microbial diversity and the microbiome was resilient to reversal after the exposure had ended. Considering that livestock farms are rich habitats for microorganisms, the various environmental microbes readily interacted with the students and modified their microbiota composition in cases where strict hygiene practices were lacking. Thus, this kind of microbiota alteration depended on continuous exposure to the environmental microbes and was easier to revert when the host adapted to the new environment. In this regard, the microbial factors were speculated to have a stronger impact than non-microbial factors in farm B, where the microbial composition and diversity were formed along a tradeoff between microbial factors and non-microbial factors. Similar observations have been reported previously. Kraemer and colleagues found that compared with the population who had no contact with farm animals, swine farmers showed a significantly higher microbiota diversity in their noses (Kraemer et al., 2019). In the same article, the authors also indicated that both microbiota diversity and structure of ex-swine farmers became similar to those of the non-exposed populations after they completely terminated the exposure to swine. Through the comparison of data collected from farm A, farm B, and prior studies, it is conclusive that the microbiota modifications related to occupational exposure are the outcome coordinated by microbial and non-microbial factors in specific environments.

### Occupational exposure compositionally and functionally reshaped the skin and nasal microbiome

Further analysis also indicated that there were more numbers of taxonomic genera enriched after exposure in farm B than in farm A, which corresponded with our observation of elevated microbial diversity in farm B. In farm A, the *Clostridium_sensu_stricto_1* was one of the few species that significantly prevailed in the skin microbiota of the volunteers. This Clostridium genus contains *Clostridium perfringens*, which exhibit a relatively high prevalence in swine farms and are often associated with necrotic enteritis in piglets (Baker et al., 2010; Chan et al., 2012). Therefore, it is rational that this animal-related genus was enriched in the students in farm A *via* direct contact with swine or their excreta. However, the abundance of this genus did not increase in the interns from farm B. This is most likely due to the great number of other bacterial species that increased in students who were exposed to farm B, which made the change of relative abundance of this genus inconspicuous.

It has been well established that composition determines the functions of microbial communities (Wang et al., 2022). Prior research indicated that occupational exposure might reshape the functions of gut microbiota regarding nutrient catabolism, stress response, and bacterial pathogenesis (Shang et al., 2022; Xie et al., 2022). However, even though it is of high interest, little is known about how skin and nasal bacteria functionally respond to occupational exposure. With this in mind, we conducted a functional prediction on the skin and nasal microbiota. Pathways involved in metabolic capacities were the most enriched functions in skin and nasal microbiota, which is also frequently observed in gut microbiota. In comparing the impacts of being exposed to farm A versus farm B, the function related to beta-lactam resistance was predicted to increase after exposure in farm A yet decrease in farm B. As discussed above, non-microbial factors, such as thorough disinfections, were supposed to be responsible for the reduced bacterial abundance and diversity in farm A. Meanwhile, such non-microbial factors might promote the emergence and enrichment of ARGs through high-level selective pressure. For instance, there have been several studies revealing that the commonly-used chlorination disinfection methods enhanced the development and spread of antibiotic-resistant genes *via* genetic mutation and horizontal transfer (Jin et al., 2020; Ma et al., 2022). The frequent use of biocides, such as triclosan, reportedly increased the risk of antibiotic resistance, as exposure to such biocides might lead to resistance against beta-lactams *via* upregulation of the expression of antibiotic hydrolases and efflux pump (Lu et al., 2018). It was concluded by Jia and colleagues that such promotion effect on antibiotic resistance by hygiene practices was generally selective and only specific ARGs were observed to be enriched (Jia et al., 2015). It has, therefore, partially explained why beta-lactam resistance particularly accumulated after exposure in farm A. Unlike in farm A, we addressed that the microbial factors played a major role in driving the microbiome shift in farm B and increased the microbial diversity. However, the increased microbial diversity was offset by a drastic reduction of commensal Actinobacteria phylum, which was known to be more prone to harbor beta-lactam resistance than other phyla (Gibson et al., 2015). Hence, we proposed that the decreased Actinobacteria abundance during exposure in farm B might account for the observed reduction in predictive beta-lactam resistance.

## Conclusion

In conclusion, the present study depicted that occupational exposure to swine farms compositionally and functionally remodeled the skin and nasal microbiota of healthy individuals. By comparing the results from the two groups, both non-microbial and microbial factors in the occupational environments were found to actively participate in defining the skin and nasal microbiota, leading to different outcomes. The findings in this study shed light on the dynamics of skin and nasal microbiota in response to occupational exposure. Further deciphering the relevance of changes with environmental cues would be necessary to address the mechanism in-depth.

## Data availability statement

The datasets presented in this study can be found in online repositories. The names of the repository/repositories and accession number(s) can be found at: https://www.ncbi.nlm.nih.gov/genbank/, PRJNA870893.

## Ethics statement

The protocol was approved by the Institutional Review Board of South China Agricultural University (SCAU-IRB). All samples were collected under authorization from the Animal Research Committees of South China Agricultural University (SCAU-IACUC). The participants provided their written informed consent to participate in this study.

## Author contributions

HR, XL, and JS: conceptualization and supervision. KC: investigation. XW and DC: methodology, software, formal analysis, data curation, visualization, and writing–original draft. JD and CF: resources. HR, XL, and YL: writing–review and editing. All authors contributed to the article and approved the submitted version.

## Funding

This work was supported by the Guangdong Major Project of Basic and Applied Basic Research (grant 2020B0301030007), the Foundation for Innovative Research Groups of the National Natural Science Foundation of China (32121004), Local Innovative and Research Teams Project of Guangdong Pearl River Talents Program (2019BT02N054), Laboratory of Lingnan Modern Agriculture Project (NT2021006), Innovation Team Project of Guangdong University (2019KCXTD001), the 111 Project (grants D20008), and the National Natural Science Foundation of China (32002336 and 32102720).

## Conflict of interest

KC was employed by Guangxi State Farms Yongxin Jinguang Animal Husbandry Group Co., Ltd., Nanning, China.

The remaining authors declare that the research was conducted in the absence of any commercial or financial relationships that could be construed as a potential conflict of interest.

## Publisher’s note

All claims expressed in this article are solely those of the authors and do not necessarily represent those of their affiliated organizations, or those of the publisher, the editors and the reviewers. Any product that may be evaluated in this article, or claim that may be made by its manufacturer, is not guaranteed or endorsed by the publisher.

## Supplementary material

The Supplementary material for this article can be found online at: https://www.frontiersin.org/articles/10.3389/fmicb.2023.1117866/full#supplementary-material

## Footnotes

^1^ https://qiime2.org/

^2^ http://huttenhower.sph.harvard.edu/galaxy/
