# Supplementary figures and images for: Occupational exposure in swine farm defines human skin and nasal microbiota
Source: Front Microbiol. 2023 Mar 29;14:1117866. doi: 10.3389/fmicb.2023.1117866 (PMC10090692; doi:10.3389/fmicb.2023.1117866)

A

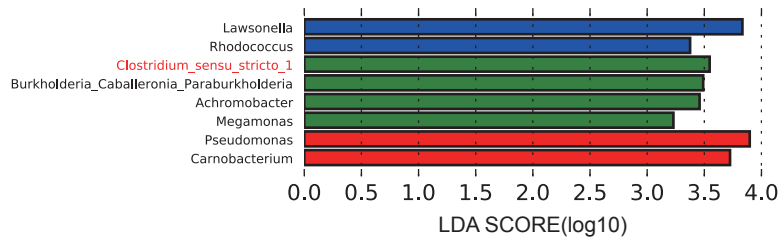

B

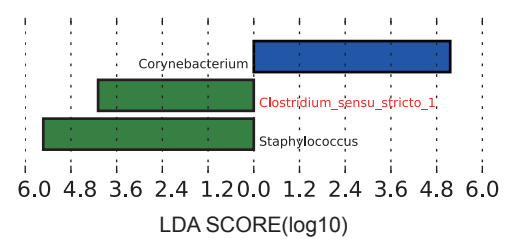

C

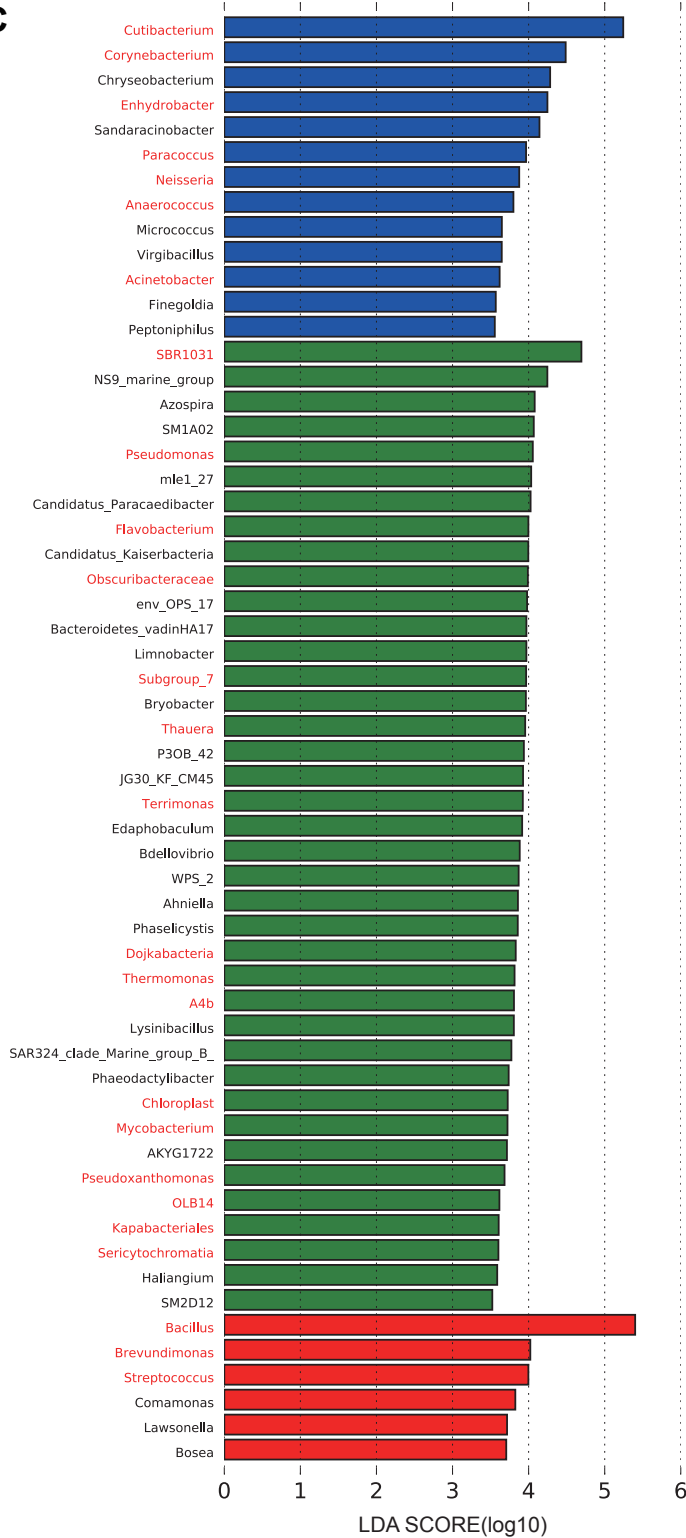

D

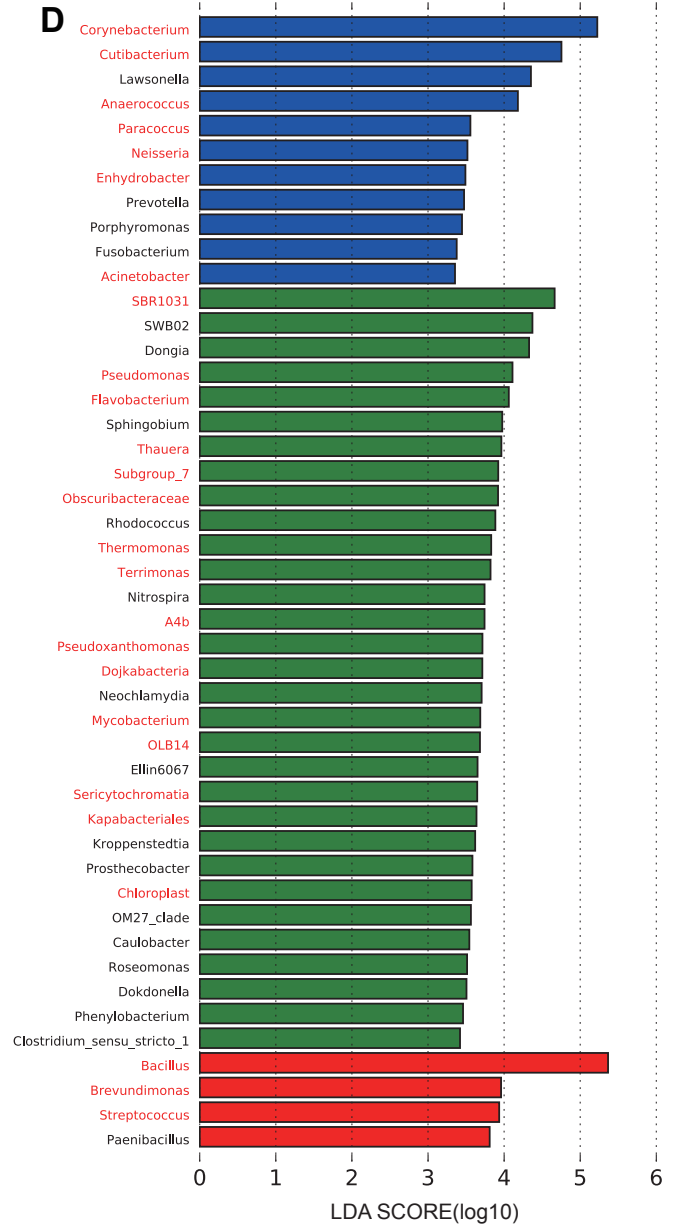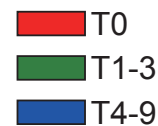

Supplement: Supplementary file 1 [file Image_1.PDF]

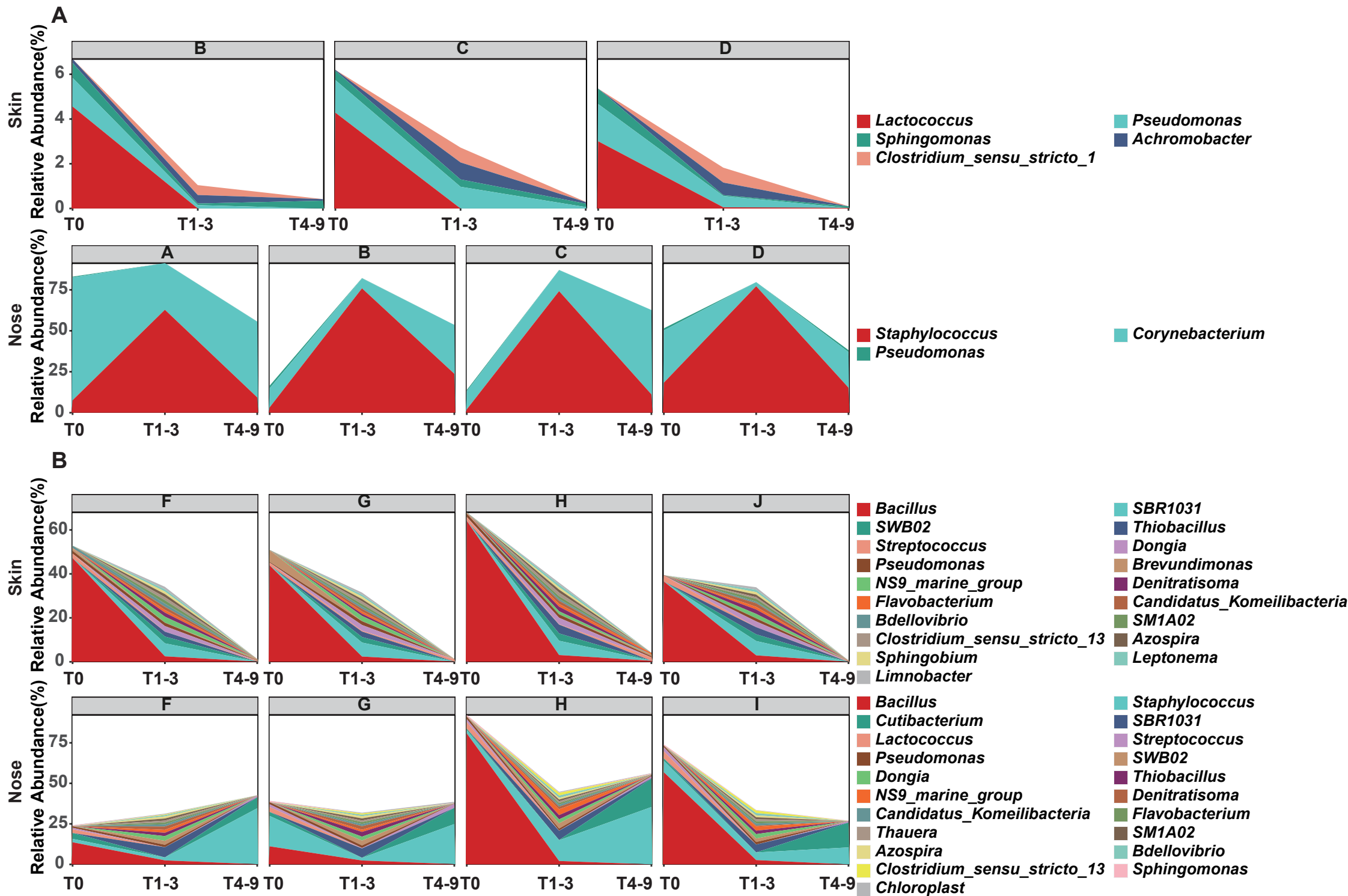

Supplement: Supplementary file 2 [file Image_2.PDF]
